# Improving quality and efficiency of trial with a novel approach to standard operating procedures (SOPS): case study from a CTU

**DOI:** 10.1186/1745-6215-16-S2-P180

**Published:** 2015-11-16

**Authors:** Diane Whitham, Lelia Duley, Alex Erven

**Affiliations:** 1 Nottingham Clinical Trials Unit, Nottingham, UK; 2 University of Nottingham, Nottingham, UK

## Background

Standard Operating Procedures (SOPs) are detailed written instructions developed to achieve consistency and high quality for a specific task or function. Frequently they are complex, confusing and intimidating for the end user, however. Nottingham Clinical Trials Unit have adapted an approach common in engineering using algorithms to improve usability of SOPs. This presentation will report development and implementation of this new system.

## Methods

Each SOPs is a simple flowchart, highlighting key steps in a process. This is supplemented with a suite of templates, forms, checklists and work instructions listed. The flowchart helps staff visualise the process, and access the appropriate supplementary materials easily and quickly. SOPS were developed with engagement from all staff in the CTU, each SOP being developed in first draft by a small working group with oversight from the lead developer.

## Results

28 SOPs have been developed and released, and this will be presented with examples of key SOPs. Development took over one year, but taking time to engage all staff in the development process has meant implementation has been rapid. Staff training is simpler to implement and keep up to date, and standardisation of common processes across the full programme of trials is improved.

## Discussion

Simple flowcharts for SOPs can be implemented in a CTU. Our experience is that development takes time, but has considerable benefit. Implementation has been rapid, and the system is popular and easy to use. Next steps are developing bespoke training, and evaluation of adherence with processes and impact.

